# Biology and Life Stages of Pine Spittle Bug *Ocoaxo assimilis* Walker (Hemiptera: Cercopidae)

**DOI:** 10.3390/insects11020096

**Published:** 2020-02-01

**Authors:** Raquel Cid-Muñoz, David Cibrián-Tovar, Ernestina Valadez-Moctezuma, Emma Estrada-Martínez, Francisco Armendáriz-Toledano

**Affiliations:** 1 División de Ciencias Forestales, Universidad Autónoma Chapingo, Carretera Federal Mexico-Texcoco Km 38.5, Texcoco 56230, Mexico; raquel.cid3004@gmail.com (R.C.-M.); dcibrian48@gmail.com (D.C.-T.); evaladezm@chapingo.mx (E.E.-M.); 2 Departamento de Fitotecnia, Universidad Autónoma Chapingo, Carretera Federal Mexico-Texcoco Km 38.5, Texcoco 56230, Mexico; nestty56@yahoo.com.mx; 3 Colección Nacional de Insectos, Departamento de Zoología, Instituto de Biología, Universidad Nacional Autónoma de México, Cto. Zona Deportiva S/N, Ciudad Universitaria, CDMX 04510, Mexico

**Keywords:** life cycle, voltism, pine decline, forest insect, nymphal instars

## Abstract

The first records of outbreaks of the Pine Spittle bug *Ocoaxo assimilis* Walker were recently identified from Puebla, Mexico, which promoted more than 2600 ha of forest foliar fall. Beyond the taxonomic and distribution information of this species, the basic traits of its biology remain unknown. This study aims to describe some biological aspects of *O. assimilis,* in a natural pine forest at Nicolás Bravo, Puebla (NB). Using morphological characteristics and a phylogenetic analysis of a fragment of cytochrome oxidase subunit I mtDNA (COI), immature instars with adults were studied; the instar number was determined by means of a multivariate analysis of 19 morphological characteristics of 121 specimens. The systematic sampling to evaluate the occurrence of nymphal specimens during a year, plus host selection experiments, allowed for determination of the abundance over time, voltism, and host preferences. Phylogenetic analysis of the COI supported that both nymphs and adults collected in NB correspond to *O. assimilis*. Principal coordinate analysis supported the existence of five nymphal stages. Field sampling and host selection experiments indicated that this species displays a single generation per year, is associated with the rainy season, and that specimens from the three first nymphal stages feed on roots of eight host species (one grass, four herbaceous species, one bush, and two trees). From the fourth instar, the insects feed on pine roots to complete their development, and when they are adults, they migrate to needles of young or mature pine stands of *Pinus pseudostrobus* to feed and reproduce.

## 1. Introduction

The *Cercopidae* species, also commonly named spittle bugs, are the largest xylem-sap sucking insect group in the world [1]. The nymphal states of this family produce copious amounts of frothy protective coverings, consisting of a mixture of air and organic compounds, that repel predators, avoid parasitism, and protect the insects from adverse weather conditions [2,3] while they obtain sufficient nutrients from the dilute xylem sap of their host [4]. These insects feed on a great variety of plants, most of them herbaceous monocots [5], herbaceous dicots [6], flowering trees [7], shrubs [8], and few species of conifers [9]. The feeding sites include leaves, branches, crowns, and exposed roots, where both nymphs and adults produce water stress and chlorosis that spreads from the feeding site, among other consequences [10,11]. In America, more than 400 valid spittle bugs species have been recorded [5]. The neotropical cercopids are best studied for the damage caused by direct feeding to forage grasses and sugarcane [12]. As a consequence of this, a detailed biology of the cercopid species is described in greater depth in taxa of economic importance [13,14,15,16,17,18,19,20,21,22,23,24]; meanwhile for most members, this topic is poorly studied, particularly in those species with conifer feeding preferences [24].

Cercopid adults have been documented feeding on conifer species of the Pinacea and Cupresaceae families; for example, *Haematoloma dorsatum* Ahrens is considered a generalist pest from Europe that promotes needle drying for at least seven *Pinus* species and also members of *Abies* Mill., *Cedrus* Trew, *Cupressus* L., *Juniperus* L., *Picea* Link, and *Pseudotsuga* Carriére [9,25,26]. The pine spittle bug *Aphrophora cribrata* [27] is another generalist species, native to North America, which causes serious injury to conifers of all sizes [28], including at least 17 species of *Pinus* L., 12 of *Picea* (Link), and three of *Abies* Mill., *Larix* Mill. *Pseudotsuga* Carriére, and *Tsuga* Carriére. Both species *H. dorsatum* and *A. cribrata* are univoltine and display five nymphal states, which feed on new leaves and roots of different species of herbs and grasses preferentially. Their adults feed on old or new pine needles and transmit microorganisms that promote the weakening of trees, which makes them susceptible to other pests and diseases [28,29].

In the last ten years, the first records of spittle bug outbreaks associated with conifers were documented in Mexican pine forests from Puebla, Oaxaca, and Veracruz States [30,31]; the symptomatology of insects was named “pine decline”, and it was similar to those caused by adults of pine spittle bug *H. dorsatum quinquemaculatum* Germar, *H. dorsatum lugens* Horvath, and *Aphrophora cribrate* (Wlk.)*,* which induces yellowish or brownish discoloration rings on the needles, resulting in its drying and falling in summer [28,29]. The impact of damage from spittle bug outbreaks ranged approximately from 2500 ha in Veracruz State [31] to 3000 ha in Puebla [32,33], which has led to the consideration of these insects as pests of economic importance in these regions. The associated agents with these damages were identified as three species of *Ocoaxo* genus [34]: *O. assimilis* Walker, *O. varians* Stål and a new species recently described *O. cardonai* Castro-Valderrama, Carvalho and Valdez Carrasco [24].

Of these species, outbreaks of *O. assimilis* were recorded in the Nicolas Bravo municipality in Puebla [30] and in the Xoxocotla, Atlahuilco, Soledad Atzompa, and Tequila municipalities in Veracruz [31]; areas where *Pinus pseudostrobus* var (Lindl.) Martínez, *P. patula* Schiede ex Schltdl. and Cham. and *Quercus rugosa* Née, predominate [35]. In both the Puebla and Veracruz states, this spittle bug displayed the symptomatology of “pine decline” and more than 2650 ha of pine forest presented foliar fall [31]. The records in entomological collections and photographic records in the naturalist database, also support the presence of this species in some municipalities from Oaxaca State [36,37].

Beyond the taxonomic and distribution information of these species promoted by recent outbreaks, the most basic aspects of its biology remain as a question to be answered. The nymphal stages of *O. assimilis* and other *Ocoaxo* species are undescribed and several basic aspects of their biology are unknown such as: the number of generations per year (voltism), reproduction season, nymphal habitus, nymphal feeding spectrum, number of nymphal instars, etc. The absence of biological information of *Ocoaxo* species has led some to reconsider their relationship with pine decline [30]; therefore, its status as a pest species of economic importance is questioned.

This study aims to describe some biology traits of *O. assimilis* in a natural pine forest at Nicolas Bravo, Puebla, Mexico. The focus is to elucidate basic aspects of the habits and behaviors relevant to its pest status, including association of nymphal stages with adults, description of nymphal instars, and preferences of the life stages, voltism, and life cycle biology. The results will further our understanding of the variation in the biology and behavior of this group and will guide improvements in spittle bug management based on species and life-stage-specific understanding.

## 2. Materials and Methods

### 2.1. Study Site

This investigation focused on a population of *Ocoaxo assimilis* located in the municipality of Nicolas Bravo, Puebla, Mexico, where there have been reports of damage to pine forests caused by this species since 2008 [30]. This region presents the temperate sub humid climate, with lower humidity rains in the summer; found between 1860 to 2800 m a.s.l. where the Pine-Oak forests predominate [38]. The samplings were carried out in the “Las Majaditas” property, which is located northwest of the municipal capital; in this area, a polygon of 42 hectares was established, corresponding to an outbreak of *O. assimilis* reported in 2017 [32], where *Pinus pseudostrobus* var *apulcensis* (Lindley) Martínez and *Quercus rugosa* Née inhabit.

**Figure 1:**
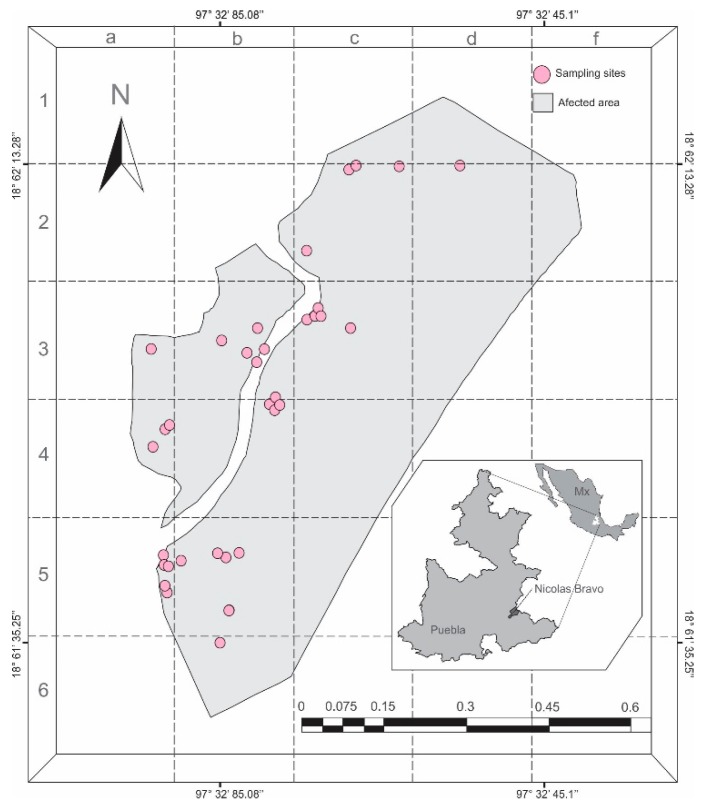
Map of sample sites of *Ocoaxo assimilis* in Nicolas Bravo, Puebla. Each pink circle represents an area, where a quadrant of 9 × 9 m was sampled.

To establish the monitoring zones, on the map of the study area, a layer with 30 quadrants of 200 × 200 m was superimposed (Figure 1). Two field inspections were conducted per month; the frequency of sampling increased to eight per month once the first nymphal stage was found. At each visit, a random quadrant was selected, in which a smaller square of 9.0 m^2^ was established in sites with pine trees that presented chlorotic spots, a most evident symptom of damage by *O. assimilis* [30]. Within the square, two people performed the active search for saliva masses containing nymphs, in the soil and roots of herbaceous plants, grasses, and trees; and the adults were collected on pine needles. Putative nymphal and adult insects of *O. assimilis* were collected live and in absolute alcohol over one year between the months of December 2017 and January 2019.

### 2.2. Specimen Identification

The morphological identification of adults was carried out using the key of Castro-Valderrama et al. [24] from external and male genital morphology. As, adults of *O. assimilis* have not been associated with nymphal stages [30], a fragment of cytochrome oxidase subunit I mtDNA (COI) was amplified from two adults identified morphologically and for specimens of the different nymphal stages, to evaluate their position in a phylogenetic tree including other Cercopid and *Ocoaxo* species. The DNA extractions of the nymphs and adults were conducted using the standard protocol for Kit EZ-10 spin column genomic DNA (Bio Basic Inc.). Molecular protocols for amplification of a 970 bp fragment of COI followed those of Paladini et al. [39]. Sequences were edited manually, concatenated and aligned using Seaview 2. 2 [40] and ClustalX 1. 83 [41].

The sequences of *O. assimilis* (KX239960.1), *O. lineatus* Walker (KX239961.1), *O. ornatipenis* Stål (GU446994.1), *O panamensis* Nast (KX239962.1), *O. tucurricae* Lallemand (GU447030.1), *Hybosgarta melichari* Lallemand (KX239946.1), *Sphenorhina distinguenda* Walker (KX239967.1), *S. coronata* Lallemand (KX239966.1), *S. parambae* Jacobi (GU4444.1), *S. rubra* Linné (GU447015.1), *S*. *prosiherpina* Distant (GU447011.1) from Paladini et al. [39] were included in the analyses; those of *H. melichari* and *Sphenorhina* spp. were used as external groups. The maximum-likelihood (ML) algorithm implemented in the program aLRT-PHYML [42] was used to infer the phylogenetic relationships among mitochondrial DNA sequences. An appropriate model of DNA evolution and model parameters, using the Akaike information criterion (AIC) tests, were determined in Modeltest v3.7 [43]. The approximate likelihood ratio test (aLRT; [44]), with the Shimodaira–Hasegawa-like procedure option, was used as a nodal support value. The genetic distances of Nei, between and within species, were calculated in MEGA 5.2.2 [45].

### 2.3. Number of Instars

Representative specimens of the nymphal sampling period were randomly selected to determine the number of instars. Discrimination among instars of spittle bugs has been performed from the frequency distribution of continuous characteristics and the respective recognition of additional discrete attributes of the body [19,46,47,48]. Therefore, to determine the number of instars in *O. assimilis,* 15 continuous and four discrete characteristics from external morphology were evaluated on 116 nymphal specimens.

For this, specimens were placed in 80% glycerin solution for three hours, to avoid dehydration and allow mobility. Appendages (legs and depending on the nymphal instar, wing pads or wings) were retired from all specimens to correct positioning and measuring. Images of body (dorsal and ventral view), right wing pad or wings (I) and right legs (I-III) from all specimens were obtained. A subset of specimens were examined with a Hitachi S-2469N scanning electron microscope (SEM) for further refinement of initial observations. The continuous characteristics were measured from the images using discrete homologous anatomical loci or landmarks (lm); which were located in a two-dimensional space by Cartesian coordinates in the program tpsDig ver. 2.31 [49]. Landmarks (lm’s) were placed to define the length and width of appendages and different parts of body. Measurements were obtained in micrometers from landmark configurations, measuring the distance of the respective lm´s in PAST ver. 1.95 [50].

Character states for each of the following attributes were documented for all individuals:

*1. Body Color (BC).* (1) Hyaline, (2) white to cream, or (3) reddish-orange. Nymphal states display conspicuous changes in color body through development [19,30].

*2. Number of flagellomeres (NF).* The multiannulated flagellum of an antenna is divided in different number of “segments” called flagellomeres; the number of these segments increase through development in cercopid species [19].

*3. Development state of wings (SWD).* In dorsal view. (0) without wing appendages, (1) wing pads do not exceed thorax, (2) wing pads do not exceed second abdominal segment, (3) wing pads exceed third abdominal segment, or (4) wings longer than abdomen. The encased undeveloped wings in the hemimetabola nymphs are called wing pads [51]. The presence and relative length of these appendages varies through development [19].

*4. Number of metatarsi spines (NMS).* The tarsomeres in some cercopids adults display cuticular ornamentations like spines, the number of these elements increase through development [19].

*5–19. The next continuous characteristics were measured*: 5) body length (BL), 6) length of cephalic capsule (LCC), 7) width of cephalic capsule (WCC), 8) distance between eyes (DE), 9) stylet length (SL), 10) thorax length (TL), 11) abdomen width (AW), 12–14) femur length from first (FI), second (FII), and third (FIII) pair of legs, 15–17) tibia length from first (TI), second (TII), and third (TIII) pair of legs, 18–19) length of right anterior wing pad or wing (LRWa) and width of right anterior wing pad or wing (WRWa).

#### Statistical Analyses of Morphological Characteristics

The normality of the distribution of each continuous characteristic was tested independently by the Shapiro–Wilkinson test [52]. To evaluate if the variation of these characteristics display discontinuities that allow the identification of discrete groups corresponding to instars, distribution histograms were plotted to each one. Additionally, to explore multidimensional patterns of morphological variation of both continuous and discrete characteristics to recognize the nymphal instars, a principal coordinates analysis (PCoA) was performed considering each specimen as an operational taxonomic unit (OTU). This was computed from a Gower distance matrix, calculated using the 15 continuous plus four discrete characteristics defined in this study [53]. To analyze the individual contribution of continuous and discrete characteristics to discriminate among nymphal instars and adults, the minimum, maximum, mean, and standard error for each continuous attribute were calculated and the Kruskal Wallis test, with respective Tukey and Mann–Whitney test comparisons were performed [54].

### 2.4. Voltism, Life Cycle and Host Spectrum

The period of occurrence of *O. assimilis* was evaluated from the field inspections; at each visit, the number of saliva masses was recorded within the quadrants. All saliva masses found within the quadrant were collected for the posterior determination and quantification of nymphal instars contained within them. Climatological variables, average monthly rainfall (avg R), and monthly average minimum temperature (avg min temp) were obtained from the databases of the National Water Commission (CONAGUA, acronym in Spanish) [55]. To determine the host species of nymphal instars of *O. assimilis*, when the saliva masses were detected on the roots, they were followed to find the aerial part of the plant, which was collected for later identification. Nymphs were preserved with 96% alcohol for posterior analysis. With the data obtained in the field, a matrix was constructed in which, for each quadrant sampled, the absence/presence of instar stages and the host plants in which they were found, were documented.

*Host Preferences*. Saliva masses of the first tree nymphal instars (NI–NIII) were found feeding in eight host plants: one grass, four herbaceous species, one brush, and two arboreal species (see results); meanwhile, nymphal instars of N4, N5, and adults were recorded in arboreal species. Thus, two experiments were performed, one of them to determine the feeding preferences of the NI, NII, and NIII stages, and the second to evaluate if the arboreal species where saliva masses of NIV, NV, and adults were found correspond to hosts in which the insect can develop its metamorphosis (nymph-adult).

**Figure 2:**
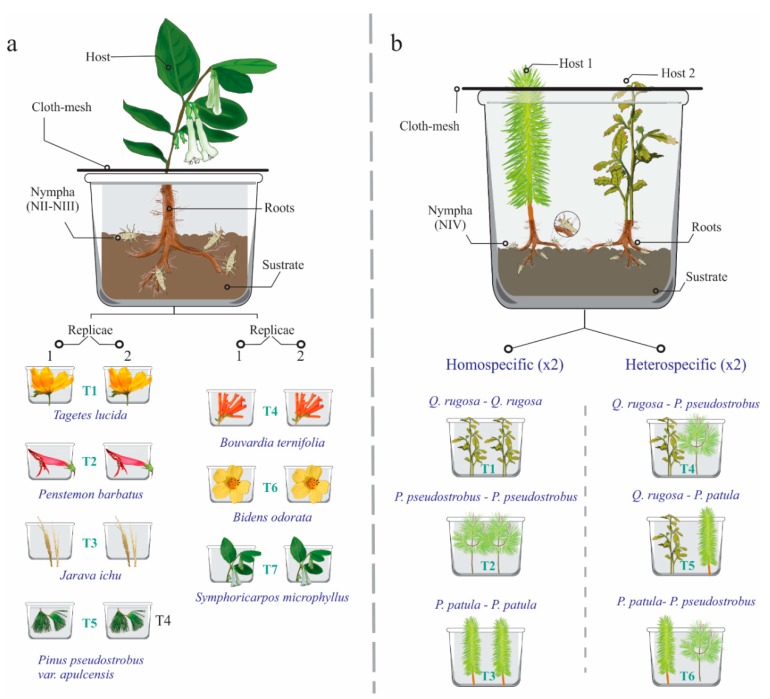
Diagrams of the two experiments to evaluate host preferences of *Ocoaxo assimilis*; in both experiments, substrate from the same sampling site, host plants, and insects were put inside of plastic boxes. **a**) Experiment 1: seven treatments were considered (T1–T7) corresponding to seven host species, respectively, each of them was carried out in duplicate (two plants per species); in each box a plant and four insects from nymphal stage one (NI) to three (NIII) were included, for a total of 14 host plants and 56 insects. **b**) Experiment 2: six treatments were considered (T1–T6) corresponding to six homo and hetero specific combinations of three hosts of tree species, each treatment was performed in duplicate; in each box, two plants and eight insects from nymphal stage four (N4) were included, in total, 14 host plants and 96 insects were studied.

In the first experiment, under controlled conditions, young plants of each host species were selected and transplanted to plastic boxes with substrate from the same sampling site. The containers with the plants were kept in a conditioned place near the study area, to monitor them. In each plastic box, a host plant and four insects collected during the first half of July, a period in which nymphs were observed in early stages (N2–N3, see results), were put inside. A total of 14 boxes, corresponding to seven host species (two boxes per host species = two biological replicas) and 56 insects were analyzed (Figure 2a). Every third day, the containers were sprinkled with water to maintain humidity and the number of saliva masses present, used as an indicator of live nymphs, was recorded. After one month, the percentage of survival of the nymphs in each host species, including both replicates, were counted. Host specimens were deposited in the herbarium of the Forest Sciences division of the Universidad Autónoma Chapingo (Numbers: 69559 to 69565).

In the second experiment, walking tests to paired selections of tree hosts and the respective development of the last nymphal stages were performed under controlled conditions. Thus, young plants of *Quercus rugosa* Née, *Pinus pseudostrobus* var. *apulcensis* and *P. patula* Schiede Ex Schltdl and Cham were placed in plastic containers (90 × 45 × 20 cm). In each box, two trees were placed with the same substrate from the sampling site; they were separated by 15 cm of distance between them (Figure 2b). Two specimens corresponding to the same host species and inter-specific combinations of the three hosts were placed in each plastic container, quantifying a total of six treatments: (T1) *Q. rugosa—Q. rugosa*, (T2) *P. pseudostrobus* var. *apulcensis—P. pseudostrobus* var. *apulcensis*, (T3) *P. patula— P. patula*, (T4) *Q. rugosa—P. pseudostrobus* var. *apulcensis*, (T5) *Q. rugosa—P. patula,* and (T6) *P. pseudostrobus* var. *apulcensis—P. patula*; each treatment was performed in duplicate. Although *P. patula* was not recorded in the study site, this host was included in the experiment as it is distributed around the study area and there are records of *O. assimilis* feeding in this species [31]. Within each box, the roots of both trees were carefully washed until their surface was uncovered from 30% to 50% (Figure 2b).

Later, eight specimens of nymphal stage four (N4) were placed in each box (four individuals per tree). In each host specimen, the insects were placed 10 cm away from the root. The boxes corresponding to each treatment were placed in a Prendo CB-20 bioclimatic chamber, controlling the humidity and temperature of the soil with KC-300 4 in 1 Soil Survey Instrument sensor, as well as the internal temperature of the chamber, programming it to a temperature range between 18 and 21 °C. The treatments were monitored daily and the number of saliva mases and adults present were counted, the experiment was followed until the last adult emerged.

In both experiments, the formation of protective frothy excreta and the development of specimens of these stages on the roots of these plants were interpreted as a signals of host selection and establishment of them for feeding and development, respectively.

## 3. Results

### 3.1. Identification of Specimens

**Figure 3:**
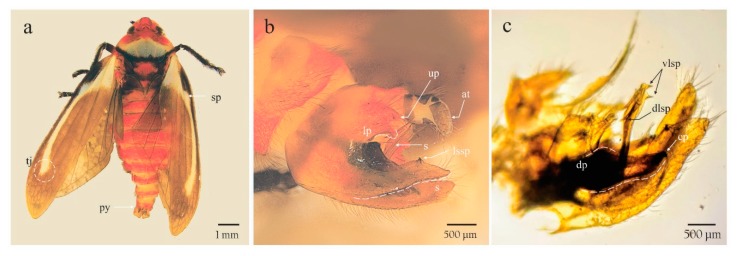
Male adult of *Ocoaxo assimilis*. **a**) dorsal habitus showing a basal spot joined to a cream yellow longitudinal line (sp), **a**) poorly delineated “tajamata” (tj) on both alar appendages and pygidium (py); **b**,**c**) pygofer in lateral view showing anal tube (at), lower process of pygofer (lp), lateral spine of the sub genital plate (lssp), shaft (s), claw of the paramere (cp), dorsal process (dp), dorsolateral spines of aedeagus (dlsp), and ventrolateral spines of aedeagus (vlsp).

The adults showed a dark brown to black spot in the union of the postclypeus and in the mandibular plate that is born in the antennal fossa; tegmina with a cream and yellow basal spot joined to a cream and yellow longitudinal line, ending as a poorly delineated “tajamata”, dark brown wing background (Figure 3a); male genitalia with parameres strongly curved, forming a right angle before the tip; subgenital plate broad and short, dilated apically; aedeagus shaft, its distal third slightly curved in lateral view, slightly thinner toward the base in ventral view; and dorsolateral spines of aedeagus straight and away to the shaft (Figure 3b,c). These characteristics correspond with those described as diagnostic from *Ocoaxo assimilis* [24].

Eight partial sequences, one of them corresponding to an adult identified morphologically as *O. assimilis* and seven to different nymphal states were obtained. Double peaks, nonsynonymous mutations, indels, frameshifts, and additional stop codons were not observed, which suggest a low probability of having sequenced nuclear copies (nuclear mitochondrial DNA (NUMTs)). After the manual edition of the sequences, a fragment of 970 bp was used in the analysis. All obtained sequences correspond to positions between 1667 and 2639 in the mitochondrion genomes of Cercopid species *Cosmoscarta bispecularis* White (KP064511.1)

**Figure 4:**
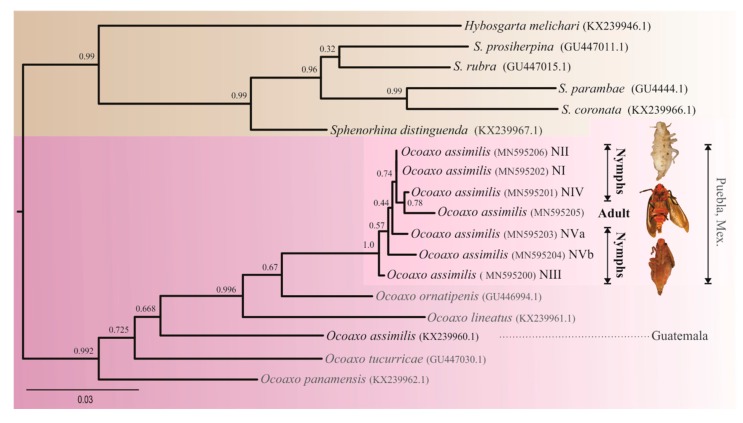
Phylogenetic tree resulting from ML analysis of 970 bp cytochrome oxidase I (COI) sequences, obtained from both nymphal and adult specimens of *Ocoaxo assimilis* and other Cercopids. The model with the best fit for nucleotide substitution was TIM2+I+G (—LnL = 3937.7517; AIC = 7962.5, K = 42 G = 0.224). Support values at nodes were derived from 1000 pseudo replicates. Nymphal stages one to five (NI, NII, NIII, NIV, NVa, and NVb).

The recovered phylogenetic tree showed a clear separation among the specimens studied and other *Ocoaxo* species (Figure 4). All nymphal sequences were clustered together with the sequence from adult specimens, within a single group with high nodal support (100%). The average genetic distance among sequences of this cluster was 0.00305, which supports that nymphal specimens correspond to the same species as the adults identified as *O. assimilis* (Figure 3a). However, the target sequences of *O. assimilis* from Nicolas Bravo of this study displayed considerable differences with respect to the sequence of *O. assimilis* from Guatemala previously deposited in the National Center for Biotechnology Information (NCBI) [39]. Our sequences were not clustered together with *O. assimilis* sequences from Guatemala, rather with *O. ornatipenis* and *O. lineatus,* respectively; the average genetic distances among target *O assimilis* sequences respect to its conspecific from Guatemala was greater (0.08467) with respect to the other species *O. ornatipenis* (0.04858) and *O. lineatus* (0.0766).

### 3.2. Number of Instars

Four outliers from the total data were excluded from the analyses in each of the 15 continuous morphological characteristics (n = 121). Without outliers, only two variables were normally distributed (LCC and AW), while 13 were not (BL, WCC, DE, SL, TL, FI, TI, F2, TII, FIII, TIII, LRWa, and WRWa) (Table 1). Distance between eyes (DE), thorax length (TL), femur length from third pair of legs (FIII), and tibia length from third pair of legs (TIII) were skewed to the right; meanwhile body length (BL) and stylet length (SL) were skewed to left. Width of cephalic capsule (WCC) and femur, tibia, and wing measurements (FI, FII, TII, TII, LRWa, and WRWa) showed bimodal distributions.

**Figure 5:**
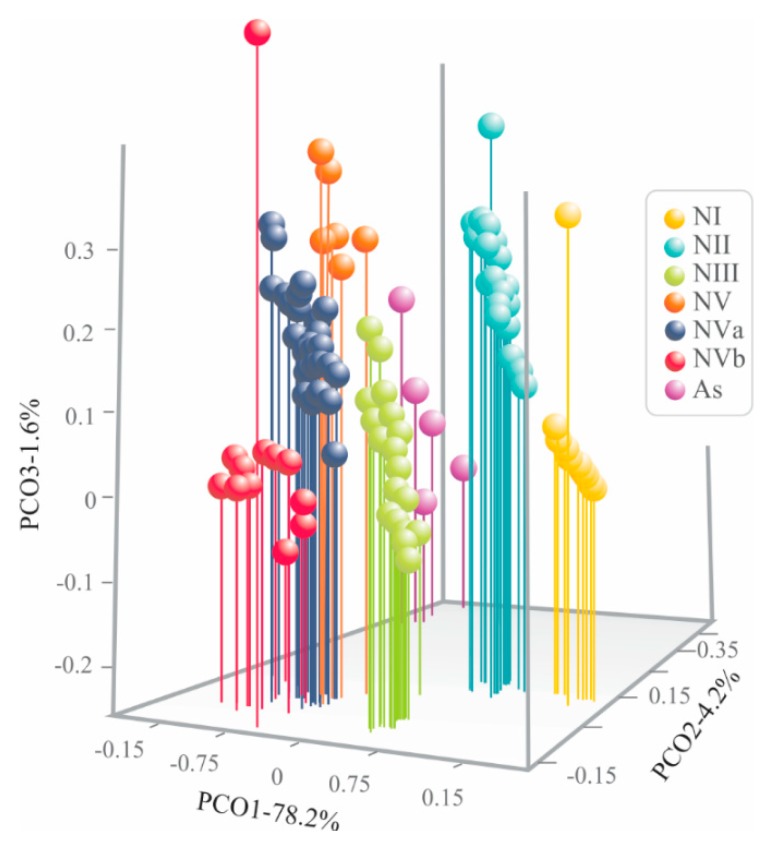
Scatter plot from principal coordinate analysis of morphological characteristics of both nymphal and adult specimens of *Ocoaxo assimilis* using 15 continuous, plus four discrete, characteristics. Adults (As) and nymphal stages one to five (NI, NII, NIII, NIV, NVa, and NVb).

The first three coordinates of the PCoA considering 15 continuous characteristics, plus the four discrete characteristics (BC, FN, SWD, and SMN) from both nymphal and adult specimens, quantified 84% of total variation (PCo 1: 78.2%, PCo 2: 4.2%, PCo 3: 1.6%). The scatter plot of the three first coordinates (PCo 1, PCo 2, and PCo 3) showed segregation of the specimens into seven well recognizable discrete phenotypic groups (Figure 5), one of them corresponding to adults (AS) and the remainder to nymphal stages (NI–NVb). The groups corresponding to nymphal specimens were segregated in PCo 1 and PCo 3, meanwhile adults were along PCo 2.

The six nymphal groups recovered by PCoA in *O. assimilis* correspond to the five nymphal instars recognized in Cercopiodea, with the last instar was subdivided in 5a and 5B (see discussion; Figure 5).

#### 3.2.1. Relative Contribution of Characteristics

Discrete frequency distributions were not found in any continuous characteristics among five nymphal instars recovered by multivariate analysis. The maximum and minimum values were overlapped between contiguous nymphal states (Table 1). However, the Kruskal–Wallis and Tukey´s tests supported that 15 continuous characteristics display differences among groups of at least three or four nymphal states, however these were not always presented in the same groups (Table 1). The specimens of nymphal state one (N1) showed lower average values in 15 characteristics (BL, LCC, WCC, DE, SL, TL, AW, FI, TI, FII, TII, and FII) than the specimens of other instars and adult state (At); the first three instars (NI–NIII) displayed differences among them in FII, TII, FIII, and TIII attributes. The average values of these instars were lower than NIV, NV, and At. NII and NIII did not show differences in ten attributes (BL, LCC, WCC, DE, SL, TL, AW, FI, TI, and TIII). Average values in these instars were higher than NI and lower than NIV, NV, and As. Nymphal states NIV, NV, and As presented similar average values in ten measurements (WCC, DE, SL, TL, AW, FI, TI, FII, TII, FIII, and TIII), which were higher than NI, NII, and NIII. The last two instars were differentiated by their higher average values in LRWa and WRWa.

**Figure 6:**
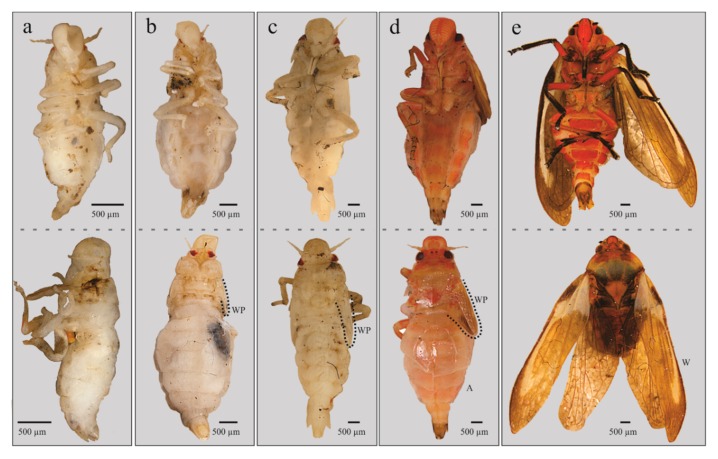
Immature stages of *Ocoaxo assimilis* in ventral (up) and lateral (down) views, corresponding to nymphal stages three (NIII) to adult (As): **a**) NIII, **b**) NIV, c) NVa, **d**) NVb, and **e**) As. A (abdomen), W (wing), and WP (wing pad).

**Figure 7:**
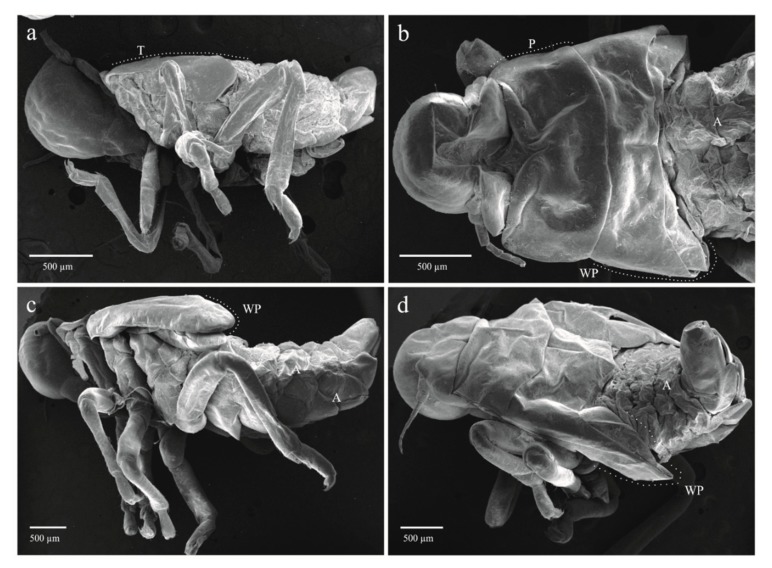
Immature stages of *Ocoaxo assimilis* showing different degrees of development of wing pads. **a**) Nymphal instar one (NI) in lateral view, **b**) nymphal instar three (NIII) in dorsal view, **c**) nymphal instar four (NIV) in lateral view, **d**) nymphal instar five “a” (NVa) in posterior-dorsal view. A (abdomen), P (pronotum), WP (wing pad).

**Figure 8:**
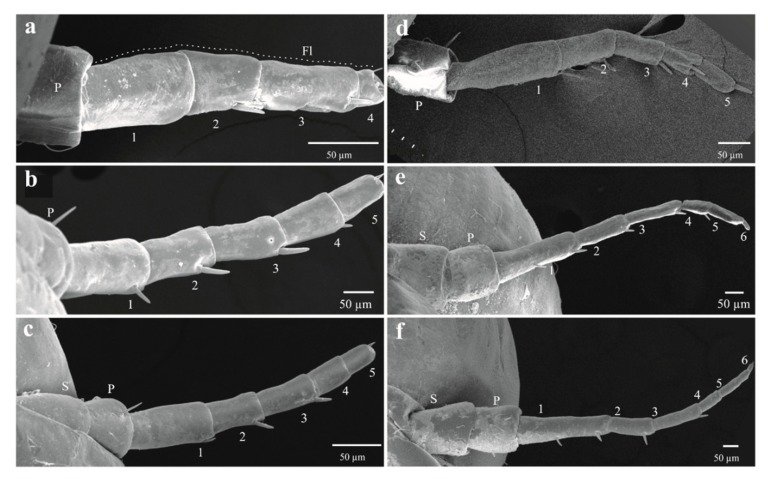
Antennae of the immature sages of *Ocoaxo assimilis*, corresponding to nymphal instar one to five (NI–NV). **a**) NI. **b**) NII, **c**) NIII, 21) NIV, **d**) NVa, and **e**,**f**) NVb. FL (flagellum), P (pedicel), S (scape). The number in antennal segments indicate the number of flagellomeres.

The four qualitative attributes showed exclusive character states among different instars. All specimens from nymphal state one (NI) to four (NIV) displayed hyaline yellowish body color (BC) (Figure 6a, b); those from NVa presented as hyaline yellowish (Figure 6c), those of NVb, reddish-orange (Figure 6d); and adults showed a reddish-orange body (Figure 6e). The development state of the wings (SWD) was also different among nymphal states. In all specimens from instars NI and NII, the wing appendages were absent (Figure 6a and Figure 7a), in insects from NIII, the length of wing pads do not exceed the thorax (Figure 6b and Figure 7b), in NIV these appendages do not exceed the second abdominal segment (Figure 6c and Figure 7c), in NVa (Figure 6c and Figure 7d) and NVb (Figure 6d) the length exceeded the third abdominal segment, and in adults the wings were larger than the abdomen (Figure 6e). The number of antennal flagellomeres (NF) and number of metatarsi spines (NMS) increased through the nymphal states; four flagellomeres were present in NI (Figure 8a), five in NII, NIII, and NIV (Figure 8b–d), and six in NVa and NVb (Figure 8e,f). As in other Cercopids, the flagellomeres in adults are fused and form two distinct portions, an expanded basal bulb-shaped portion and a threadlike apical arista [56]; meanwhile two metatarsi spines were quantified in NI, four in NII, six in NIII, nine in NIV, and 11 to 14 in NV and adults.

#### 3.2.2. Instar Description

**Figure 9:**
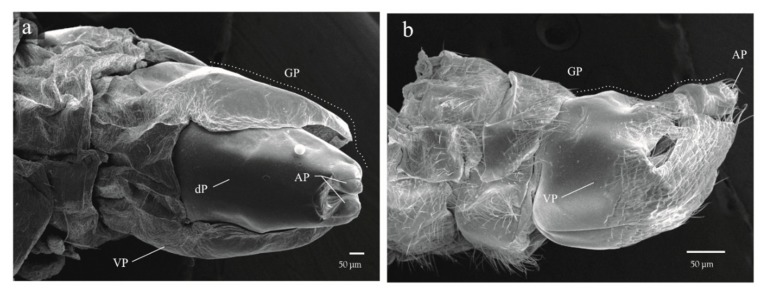
Apical portion of the abdomen of *Ocoaxo assimilis* nymphal instars with genital plates on the terminal segment developed. **a**) Male in dorsal view, showing two short apical processes with the ventral plate covering half of the dorsal plate, **b**) female in lateral view with two pairs of acute processes with the ventral plate covering most of the dorsal plate. AP (apical processes), GP (genital plate), VP (ventral plate).

Morphological differences of both continuous and discrete characteristics among nymphal stages and adults are summarized in Table 1. The five nymphal instars (NI–NV) can be reliably identified using body color (BC), number of “flagellomeres” in antennae (NF), development state of wings (SWD), and number of metatarsi spines (NMS). The specimens of the last nymphal instar (NV), displayed abdomens similar to earlier instars with the genital plates on the eighth and ninth segment more developed, allowing differentiation of the sexes (Figure 9a,b); furthermore, conspicuous morphological differences between the Va and Vb subgroups can be recognized (Table 1). In addition to the characteristics in Table 1, the wings of adults display the diagnostic tegmina with a cream-yellow basal spot joined to a cream yellow longitudinal line, ending as a poorly delineated “tajamata” (Figure 3a and Figure 6e).

### 3.3. Voltism and Life Cycle

**Figure 10:**
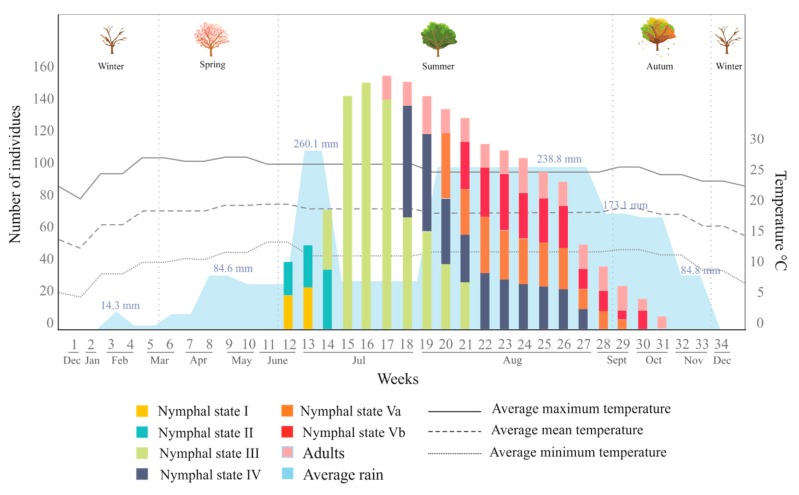
The number of individuals of *Ocoaxo assimilis* collected from December 2017 to December 2018, in Nicolas Bravo, Puebla, corresponding to nymphal and adult stages. Abundances per stages are presented in the color bars; the average monthly rainfall is shown along the bottom in blue; the average monthly temperatures (mean, maximum, and minimum) correspond to the bottom lines.

The presence of *O. assimilis* was evaluated from the last week of December 2017 to the first week of January 2019; in this period, a total of 34 quadrants were analyzed (Figure 1), in which 1612 saliva masses containing the same number of specimens were recorded. Figure 10 summarizes the number of individuals collected per stage, their occurrence through year, and the environmental variables measured in the period (rainfall and temperature).

From these records, the occurrence period of *Ocoaxo assimilis* in the pine Forest of Nicolas Bravo was documented, which showed a marked seasonality from the beginning of the rains in summer, in the second half of June, until autumn, in the second half of October. In this period, eggs were not found in the area and, in total, 1612 specimens of five nymphal instars (NI-NV) and adults corresponding to only one brood were recorded and collected.

The presences of the nymphs were synchronized with the beginning of the rainy season. The nymphal instars NI and NII were recorded from the end of June until the second week of July; which was associated with changes in the average monthly rainfall (RM) from 72.1 mm to 260 mm and a monthly average minimum temperature (TM) from 12.4 to 14 °C. The highest abundances of NI (n = 26) and N2 (n = 36) specimens were recorded in the first and second weeks of July, respectively. Nymphal instar three (NIII) was observed during the entire period of July until the first three weeks of August, overlapping with NII, this period presented 76.9 mm RM and 11.9 °C TM. The highest abundances of NIII specimens were recorded in the middle of July (n=152). Nymphal state four (NIV) was recorded from the last week of July, overlapping with NIII, to the last week of August. This period presented increases from July to August in both RM from (76. 9 mm to 238.8 mm) and TM (12.4 to 14 °C). The highest abundance of NIV was quantified in the first week of August (n=34). The last nymphal instar (NV) was collected from the second week of August to the second week of October (overlapping with NIV), the period in which there was environmental changes quantified in RM (238.8 mm in August to 173.1 mm in September) and TM (14 °C in August to 12.1 °C in September). Adults were found from the last week of July to the third week of October, overlapping with NV. The highest abundance of adults was recorded in the last week of August (N=35). Couples of copulating adults were recorded on pine leaves in September and October.

### 3.4. Host Spectrum

**Figure 11:**
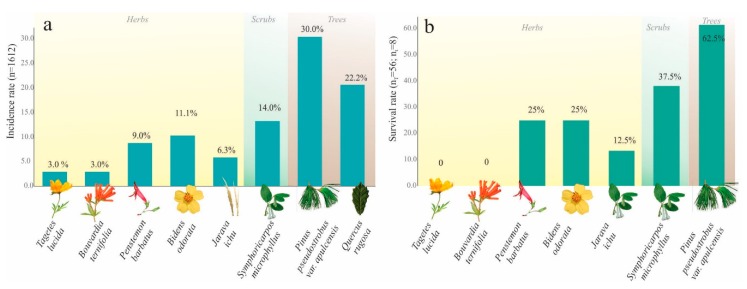
Host preferences of *Ocoaxo assimilis* in Nicolas Bravo, Puebla. **a**) The rate incidence of insects collected in the field (nymphs and adults) on the roots of eight host plants; n total number of insects collected (=1612); percentages at the top of bars were calculated with respect to “n”. Survival rates of nymphs of *O. assimilis* under controlled conditions to evaluate the feeding preferences among herbaceous, bush, and arboreal host species. nT, the total number of insects analyzed considering the seven host species (56); nt, the total number of analyzed insects per host plant (8); percentages were calculated respect to nt. **b**) The results of experiments to evaluate the paired selection of tree hosts and the respective development of *Ocoaxo assimilis* nymphal specimens from nymphal stage “S4” to adults.

Specimens of five nymphal instars (NI–NV) were detected on the roots of eight species of plants: one grass (*Jarava ichu* Ruiz and Pav), four herbaceous species (*Bidens odorata* Cav., *Penstemon barbatus* (Cav.), *Tagetes lucida* Cav., *Bouvardia ternifolia* (Cav.) Schltdl), one bush (*Symphoricarpos microphyllus*), and two trees (*Pinus pseudostrobus* var. *apulcensis, Quercus rugosa* Née). Of them, 30% (n = 486) of nymphal specimens were found on *Pinus pseudostrobus* var. *apulcensis* (Lindl.) Martínez, 22.2% (n = 358) on *Quercus rugosa* Née, 14.0% (n = 230) on *Symphoricarpos microphyllus*, 11.1% (179) on *Bidens odorata* Cav., 9.0% (n = 153) on *Penstemon barbatus* (Cav.), 6.3% (n = 102) on *Jarava ichu* Ruiz and Pav, 3.0% (n = 51) on *Tagetes lucida* Cav., and 3.0% (n = 51) on *Bouvardia ternifolia* (Cav.) Schltdl (Figure 11a).

#### Host Preferences

The feeding preferences among herbaceous, bush, and arboreal species of *O. assimilis* nymphs under controlled conditions were evaluated through July and August from NII and NIII instars. In both replicas of seven hosts species, 100% of insects (n = 56) walked toward the roots of the plants, then secreted protective frothy excreta to begin feeding and continued their development. However, the specimens were not capable of continuing their development in all host species; in total, only 23% of them (n = 13) survived until the end of the period. The hosts in which higher survival rates of nymphs were present corresponded to the species *Pinus pseudostrobus* var. *apulcensis* with an average of 62.5%, followed by *Penstemon barbatus* with 50%, the bush *Symphoricarpos microphyllus* with 37.5%, *Bidens odorata* with 25%, and *Jarava ichu* with 12.5%. In the treatments corresponding to *Tagetes lucida* and *Bouvardia ternifolia,* no nymphs survived (Figure 11b).

The walking tests and the respective development tracking were carried out for 23 days, the period in which the last nymphal specimen reached the adult stage from NIV. In this period, 60.41% of specimens (n = 58) complete the metamorphosis before 14 days. In the six treatments, 100% of the specimens (n = 96) walked toward the roots of plants, on it, secreted protective frothy excreta to begin feeding and continued their development; however only 68. 75% of them (n = 66) could complete their metamorphosis and 31.25% died (n = 30).

**Figure 12:**
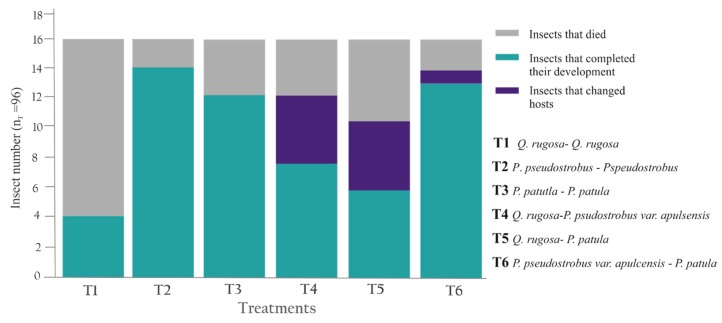
The results of the walking test to paired host tree selection of specimens of *Ocoaxo assimilis* from nymphal stages NIV. Homospecific (T1–T3) and heterospecific treatments (T4–T6).

Among homospecific treatments, T1 (*Q. rugosa*—*Q. rugosa*) displayed the lowest survival rate (25%), as 12 of the 16 insects died during the first week, before completing the metamorphosis; meanwhile T2 (*P. pseudostrobus*—*Pspeudostrobus*) and T3 (*P. patutla*—*P. patula*) showed higher survival rates, as 14 (87.5%) and 12 (75%) specimens, respectively, completed their development to the adult stage (Figure 12).

In the treatments with hetero specific combinations (T4–T6) all nymphs selected the nearest roots from where they were placed; however, during first week of the experiment some of them changed their host to a contiguous plant. In T4 (*Q. rugosa*—*P. psudostrobus var. apulsensis*) two specimens died and six completed their metamorphosis, of these last, four specimens change the tree host from oak to pine. In T5 (*Q. rugosa*—*P. patula*), six nymphs died and 10 reached the adult stage, of them, four nymphal specimens changed their host from *Q. rugosa* to *P. patula*. In T6 (*P. pseudostrobus var. apulcensis*—*P. patula*) only two insects died and seven completed metamorphosis, of which, only two changed from *P. patula* to *P. pseudostrobus* var. *apulsensis* (Figure 12).

## 4. Discussion

Despite of the recent outbreaks promoted by spittle bug species of the genus *Ocoaxo* (*O. assimilis*, *O. cardonai* and *O. varians*) in Mexico [33]; the biology of these insects were poorly described, and the nymphs and their respective hosts were unknown [57]. In the present study using morphological and molecular data we were able to associate, in *Ocoaxo assimilis,* the immature instars with adults, which in turn allowed us to determine the number of nymphal stages of this species, to know its abundance over time, and to describe the morphological changes that it presents during its life cycle. Additionally, the sampling of nymph and adult specimens, together with field and laboratory experiments, allowed us to know the voltism, spectrum, and host preferences of these stages.

*Taxonomy-identification of nymphal specimens.* Traditionally in the cercopid species, the association of nymphal stages with adults has been done under garden experiments, by means of crossing studies [48]. In other groups of insects and species of cercopids, the Barcode has constituted a reliable tool for this purpose [58,59,60,61] allowing the identification of specimens in different nymphal stages and their respective association with the adult stage, as is the case of the specimens of *O. assimilis* analyzed in the present research.

Adult insects of *O. assimilis* from Nicolas Bravo, Puebla presented diagnostic tegmina of this taxon with a cream-yellow basal spot joined to a cream-yellow longitudinal line, ending as a poorly delineated “tajamata” and aedeagus shaft with its distal third slightly curved, displaying straight dorsolateral spines. Of these characteristics, those from external morphology correspond with those included in the original description of *Ocoaxo assimilis* [27] and with the attributes displayed by the type of specimens of this species presented in Carvalho and Webb (Figure 672 in [1]). Furthermore, external and genital characteristics agree with the recent taxonomic key, provided by Castro Valderrama et al. [24], to identify *O. assimilis*.

Morphological identification, together the results of both phylogenetic analysis and calculation of genetic distances using cytochrome oxidase subunit 1 gene as a DNA barcode, supported that both nymphs and adults collected in Nicolas Bravo, Puebla correspond to a single species, *O. assimilis,* as they formed a monophyletic group and the average genetic distance among them (0.00305) was much less than those calculated among other *Ocoaxo* spp. (0.09109) included in the Genebank [39].

Outstanding results of COI analysis showed that sequences of *O. assimilis* from Nicolas Bravo, Puebla were polyphyletic, with respect to the sequence of the same species from Guatemala, previously deposited in Genebank [39]. The monophyletic group of *Ocoaxo assimilis* from Nicolas Bravo, Puebla displayed 8.2% divergence and was closer to *O. ornatipens* and *O. linneatus*, with respect to the sequence of *O. assimilis* from Guatemala. Both *O. ornatipens* and *O. linneatus* are species that have different habits and are morphologically very different from *O. assimilis* [36].

The genetic differences observed between Guatemala and Nicolas Bravo, Puebla sequences are similar to the 9.1% divergence in the COI sequence data found among other *Ocoaxo* species and 7.1% divergence in other species of Cercopidae (e. g. *Sphenorhina* spp.) [39]. The strong genetic differences observed in COI between *Ocoaxo assimilis* form Guatemala and Puebla are equivalent or higher to that found between full species *Ocoaxo*. For example, the species *O. ornatipens* and *O. lineatus* are 7.0% divergent in COI [39]. These results suggest that specimens from Guatemala and Nicolas Bravo, Puebla can be different species.

Despite that type locality of *O. assimilis* is inaccurate and it only is delimited to “Mexico” [62], several boucher specimens deposited in the Colección Nacional de Insectos, Instituto de Biología, Universidad Nacional Autónoma de Mexico (CNIN) and Colección de Insectos del Colegio de Posgraduados, Campus Montecillo (CEAM), Mexico, support the distribution of *O. assimilis* in the east of the Trans Mexican Volcanic Belt in Veracruz and Puebla states and in Sierra Madre del Sur in Guerrero and Oaxaca, states from Mexico [36]; which is consistent with the data from this study.

More integrative studies need to be done to evaluate the morphological geographical variation of this species and analyze more molecular markers to clarify the taxonomy and separate the potential species within *Ocoaxo assimilis*.

*Instar Number.* The instar number in *Cercopidae* members has been determined by mean univariate analysis using morphometric measurements, such as body length, body width, length of wings pads, length of wings, and width of cephalic capsule; of them, the most informative characteristics were the body length and the width of the cephalic capsule, as they allow the recognition of discrete groups corresponding to the nymphal stages [13,48]. Our results support that the variation of body measurements was wide and overlapped among nymphal stages, which did not allow us to separate them individually.

However, the multivariate analysis of both continuous and discrete characteristics together (PCoA), support the existence of five nymphal stages in *O. assimilis* as has been documented in other pine cercopids, such as *Aphrophora cribrata* and *Aphrophora saratogensis* [8,28]; but different from *Haematoloma dorsarum*, which presents four nymphal stages [63]. A very marked difference among *O. assimilis* and the aforementioned pine spittle bugs is that, the specimens of *O. assimilis* corresponding to nymphal state five presented morphological differences in discrete and continuous characteristics; which were recovered, in the 3D scatter plot of PCoA, as two subgroups, “Va” and “Vb” (Figure 5).

This pattern has also been identified in the grass and leaf cercopids *Mahanarva andigena*, *Zulia carbonaria*, *Aeneolamia reducta,* and *Prosapia simulans*, in which, the specimens of nymphal instar five are classified as “Va” and “Vb” based on differences in body color and size measurements [48]. The most notable differences between NVa and NVb in *O. assimilis* are body color and the presence of tegmina with a cream-yellow basal spot joined to a cream yellow longitudinal line, ending as a poorly delineated “tajamata” on the wing pads in Vb, and absent in Va. Notable changes were also distinguished throughout its development, such as development of the wing packs or stumps, which helps to differentiate the first two instars from the others. This facilitates the classification in the field of individuals per instar.

*Life cycle*. Studies of the life cycle of the cercopids have been carried out in laboratory conditions [13,19,48], which makes it difficult to associate the nymphal changes with wild environmental variables as well as to elucidate the host preferences of both nymphs and adults. Our systematic sampling to evaluate the occurrence of nymphal specimens during a year, supported that, in contrast with other tropical spittle bugs that are multivoltine [5,14], *Ocoaxo assimilis* displayed a single generation per year in Nicolas Bravo, Puebla. This is in agreement with other pine spittle bugs, such as *Aphrophora cribata*, *A. saratogensis,* and *H. dorsatum* [8,28,63,64].

The occurrence period of *Ocoaxo assimilis* displayed a notorious seasonality from the beginning of summer until the beginning of autumn. As in many cercopids, the presence of specimens corresponding to first nymphal state was coupled with the beginning of the rainy season in summer [5,8]. Most of the life cycle occurs in the summer (June to August) period, where specimens of NI to NVb stages were recorded. The remainder stages appeared gradually, and they were overlapped between themselves, from the end of summer until the beginning of autumn, which was the period in which the last NVb specimens completed their development to the adult stage. Reproduction occurs at the end of summer and beginning of autumn, the period in which the couples of copulating adults were recorded on pines principally; a behavior also described in *H. dorsatum* [29].

Although it was not possible to establish the sites and oviposition period of *O. assimilis*, as eggs were not found in the field, our results suggest that they may be deposited close to root grasses, herbs, or trees overwinter until the next spring. They then hatch in the rainy season in summer. In the red-black pine bug *Haematoloma dorsatum*, after reproduction, eggs are laid within a nick at the base of the grass and hatch in autumn; nymphs occur overwinter until the next spring.

In contrast with other pine spittle bugs where their nymphal states feed in groups of two to five individuals on the basal stem parts, roots of various grass species [63], or on herbaceous plants and woody stems [8], nymphal specimens of *O. assimilis* displayed different feeding behaviors and a wider food spectrum. They feed alone and in less frequency in pairs within different protective masses of white spittle on eight species of plants, which included one grass, four herbaceous species, one bush, and two trees. They are commonly found on the trees, *Pinus pseudostrobus* var. *apulcensis* (Pinaceae) and *Quercus rugosa* (Fagaceae); meanwhile, with less frequency, individuals were also collected from *Symphoricarpos microphyllus* (Caprifolieaceae), a shrub, followed by herbaceous plants, such as *Bidens odorata* (Asteraceae) and *Penstemon barbatus* (Plantaginaceae), all plants common in Mexican pine forests [65].

The first tree instar displayed wider feeding preferences on the eight species documented. After the third nymphal instar, the specimens from different plant hosts migrate on pine roots to complete their development. The adults migrate to needles of young or mature pine stands, where they insert the stylet into various sites on the needles selected for feeding, to this they extend out their forelegs into the air like other *Ocoaxo* species [30]. At abandoned feeding sites, typical yellowish or brownish discoloration rings on the needles appear (see Figure 2c,d in [30]); which results in the needles drying and early fall in autumn. The cause of the leaf damage is unknown; however, this is associated with the phytotoxic effects of salivary substances [30]; however, in other pine spittle bugs, damage is associated to the fungi *Sphaeropsis sapinea* [66,67]; more studies are necessary to clarify this topic.

Our host selection experiment ratifies the preference of *Ocoaxo assimilis* for *Pinus pseudostrobus* var. *apulensis* and *P. patula*, hosts in which the nymphs fulfill their metamorphosis and adults feed. Despite that, within the sampling areas, *P. patula* specimens were not found, previous records support that *O. assimilis* can infest *P. patula* in other Mexican localities [31,36].

Other pine spittle bugs with similar behavior to *O. assimilis*, such as *Aphrophora cribata*, *A. saratogensis* and *Haematoloma dorsatum* are considered as occasional pests, as they generally affect the vigor of their host tree by consuming host resources; in addition, host resources may be further depleted by indirect effects, resulting from a reduced photosynthesis due to early leaf fall [26,63]. As these species *O. assimilis* promote the drying of pine needle tips, descending to their base after the suction. The foliage becomes yellowish, orange, or brown, and then the needles fall. In an endemic event, the damages are located on disperse trees or small spots, but in epidemic conditions, this spittle bug affects a considerable forest surface. From 2014 to 2018, pine decline associated to *Ocoaxo* in Mexico quantified more than 7200 ha of pine forest, of which 26% (1886 ha) were attributed to *O. assimilis* in the Puebla and Veracruz [31]. According to our results, host trees can recover their foliage in the next spring, however, it has been documented that when this phenomenon occurs consecutively, the next years (for at least three years) many trees die [30]. It is necessary to evaluate the effects of pine decline in Mexican forests, such as changes in resource storage due to the loss of photosynthetic activity, diminution of host defense capacity of their host tree, tree mortality by effect of consecutive outbreaks by other agents, etc.

## 5. Conclusions

Our results support the existence of five nymphal stages in *O. assimilis*, with two subgroups within the fifth instar. This species displays a single generation per year (from June to October) in Nicolas Bravo Puebla and its life cycle is associated with the rainy season. Specimens of this spittle bug feed alone and in less frequency in pairs within different protective masses in roots of eight species of plants (one grass, four herbaceous species, one bush, and two trees). After the third nymphal instar, the specimens from different plant hosts migrate on pine roots to complete their development; the adults migrate to the needles of young or mature pine stands of *Pinus pseudostrobus*. Genetic distances and phylogenetic position of the sequences of *O. assimilis* from Nicolas Bravo, Puebla, with respect to the sequence of the same species from Guatemala, indicate that specimens of *O. assimilis* from Puebla and Guatemala could be different species.

## Figures and Tables

**Table 1:** Morphological characteristics analyzed from both nymphs (NI-NVb) and adults of *Ocoaxo assimilis*; basic statistics and results of the Kruskal Wallis test are displayed for 15 continuos characteristics.

	Character	NI (18)	NII (25)	NIII (20)	NIV (7)	NVa (32)	NVb (14)	Adults (5)
		(Range); Mean± SE	(Range); Mean± SE	(Range); Mean± SE	(Range); Mean± SE	(Range); Mean± SE	(Range); Mean± SE	(Range); Mean± SE
**C**	**BL^L^**	(2.1,3.6); 2.8**a** ± 0.1	(3.0,5.8); 4.2**b** ± 0.1	(3.3,5.7); 4.3**b** ± 0.2	(4.5,9.6); 5.9**c** ± 0.2	(4.5,7.2); 5.9**c** ± 0.1	(7.3,9.6); 8.1**d** ± 0.2	(7.1,9.4); 8.1**d** ± 0.4
	LCC^*^	(0.4,0.7); 0.5**a** ± 0.0	(0.6,1.0); 0.7**b** ± 0.0	(0.6,1.0); 0.8**b** ± 0.0	(0.8,1.3); 1.0**c** ± 0.1	(0.7,1.2); 0.9**c** ± 0.0	(0.7,1.1); 0.9**c** ± 0.0	(0.7,0.9); 0.8**bc** ± 0.0
	**WCC^B^**	(0.5,0.9); 0.7**a** ± 0.0	(0.7,1.3); 1.0**b** ± 0.0	(0.8,1.3); 1.0**b** ± 0.0	(1.2,1.7); 1.5**c** ± 0.1	(1.1,1.7); 1.5**c** ± 0.0	(1.4,1.7); 1.5**c** ± 0.0	(1.5,1.7); 1.6**c** ± 0.0
	**DE^R^**	(0.3,0.6); 0.5**a** ± 0.0	(0.7,0.9); 0.8**b** ± 0.0	(0.6,0.9); 0.7**b** ± 0.0	(0.8,1.2); 1.0**c** ± 0.0	(0.7,1.1); 1.0**d** ± 0.0	(0.9,1.0); 1.0**d** ± 0.0	(0.6,0.9); 0.7**b** ±0.0
	**SL^L^**	(0.3,0.7); 0.6**a** ± 0.0	(0.3,1.2); 0.7**b** ± 0.0	(0.5,1.0); 0.8**b** ± 0.0	(0.8,1.4); 1.2**c** ± 0.1	(0.7,1.5); 1.1**c** ± 0.2	(1.0,1.7); 1.4**d** ± 0.1	(1.1,1.5); 1.4**d** ±0.2
	**TL^R^**	(0.4,1.0); 0.7**a** ± 0.0	(0.8,1.5); 1.1**b** ± 0.0	(1.0,1.4); 1.2**b** ± 0.0	(1.3,2.2); 1.9**c** ± 0.1	(1.3,2.4); 1.9**c** ± 0.0	(2.0,3.2); 2.6**d** ± 0.1	(2.7,3.5); 3.1**e** ±0.2
	AW^*^	(0.6,1.3); 1.0**a** ± 0.0	(0.9,2.2); 1.5**b** ± 0.1	(1.1,2.1); 1.5**b** ± 0.1	(1.7,2.2); 2.0**c** ± 0.1	(1.6,2.5); 2.1**c** ± 0.0	(1.8,3.0); 2.4**c** ± 0.1	(2.4,3.0); 2.9**d** ±0.1
	**FI^B^**	(0.2,0.8); 0.5**a** ± 0.0	(0.6,0.9); 0.8**b** ± 0.0	(0.4,1.0); 0.8**b** ± 0.0	(1.0,1.5); 1.2**c** ± 0.1	(0.8,1.5); 1.2**c** ± 0.0	(0.8,1.4); 1.2**c** ± 0.0	(1.3,1.6); 1.5**d** ±0.0
	**TI^B^**	(0.3,0.5); 0.4**a** ± 0.0	(0.5,0.9); 0.7**b** ± 0.0	(0.6,1.5); 0.7**b** ± 0.0	(1.0,1.1); 1.0**c** ± 0.0	(0.8,1.3); 1.0**c** ± 0.0	(1.0,1.2); 1.1**c** ± 0.0	(1.4,1.7); 1.6**c** ±0.0
	**F2^B^**	(0.4,0.6); 0.5**a** ± 0.0	(0.7,1.0); 0.8**b** ± 0.0	(0.7,1.3); 0.9**c** ± 0.0	(1.0,1.4); 1.3**d** ± 0.1	(1.0,1.4); 1.2**d** ± 0.0	(1.1,1.5); 1.3**d** ± 0.0	(1.3,1.6); 1.4**d** ±0.1
	**T2^B^**	(0.3,0.6); 0.4**a** ± 0.0	(0.6,1.0); 0.7**b** ± 0.0	(0.6,1.1); 0.7**b** ± 0.0	(1.0,1.3); 1.1**c** ± 0.0	(0.9,1.6); 1.1**c** ± 0.0	(0.9,1.2); 1.1 **c** ± 0.0	(1.6,1.8); 1.7**d** ±0.0
	**F3^R^**	(0.5,0.8); 0.6**a** ± 0.0	(0.8,3.2); 1.0**b** ± 0.1	(0.8,1.4); 1.0**c** ± 0.0	(1.2,1.7); 1.4**d** ± 0.1	(0.9,1.8); 1.4**d** ± 0.0	(1.2,1.6); 1.4**d** ± 0.0	(1.3,1.6); 1.4**d** ±0.1
	**T3^R^**	(0.3,0.7); 0.6**a** ± 0.0	(0.8,1.2); 1.0**b** ± 0.0	(0.8,1.5); 1.0**b** ± 0.0	(1.5,1.7); 1.6**c** ± 0.0	(1.1,1.8); 1.5**c** ± 0.0	(1.1,1.8); 1.5**c** ± 0.0	(1.8,2.2); 2.1**d** ±0.1
	**LRWa^B^**			(0.7,1.7); 1.0**a** ± 0.1	(1.9,2.6); 2.3**b** ± 0.1	(1.5,3.2); 2.6**b** ± 0.1	(2.3,3.0); 2.7**b** ± 0.1	(8.4, 10.6); 9.4**c** ±0.4
	**WRWa^B^**			(0.3,0.8); 0.5**a** ± 0.0	(0.8,1.1); 0.9**b** ± 0.0	(0.6,1.1); 0.8**b** ± 0.0	(0.7,1.0); 0.9**b** ± 0.0	(2.8,3.2); 2.8**c** ±0.1
**D**	**BC**	hyaline	hyaline	white to cream (Figure 6a)	white to cream (Figure 6b)	white to cream (Figure 6c)	reddish-orange (Figure 6d)	reddish-orange (Figure 6e)
	**SWD**	without wing appendages (Figure 7a)	without wing appendages (Figure 7b)	wing pads do not exceed thorax	wing pads do not exceed thorax (Figure 7b)	wing pads do not exceed second abdominal segment (Figure 7c)	wing pads do not exceed second abdominal segment (Figure 7d)	wings longer than abdomen (Figure 6e)
	**NF**	four (Figure 8a)	five (Figure 8b)	five (Figure 8c)	five (Figure 8d)	six (Figure 8e)	six (Figure 8f)	fused flagellomeres
	**NMS**	two	four	six	nine	11-14	11-14	11-14

**Data distribution**: (*) Normal, (L) Left, (R) Right and (B) bimodal. **Continuous characteristics** (**C**): Body length (BL), length of cephalic capsule (**LCC**), width of cephalic capsule (**WCC**), distance between eyes (**DE**), stylet length (**SL**), thorax length (**TL**), abdomen width (**AW**), femur length from first (**FI**), second (**FII**), and third (**FIII**) pair of legs, tibia length from first (**TI**), second (**TII**), and third (**TIII**) pair of legs, length of right wing pad or wing from anterior (**LRWa**) and width of right wing pad or wing from anterior (**WRWa**). **Discrete characteristics** (**D**): body color (**BC**), number of flagellomeres (**NF**), development state of wings (**SWD**), number of metatarsi spines (**NMS**).
